# Precision Mental Health and Data-Informed Decision Support in Psychological Therapy: An Example

**DOI:** 10.1007/s10488-023-01330-6

**Published:** 2023-12-15

**Authors:** Wolfgang Lutz, Jana Schaffrath, Steffen T. Eberhardt, Miriam I. Hehlmann, Brian Schwartz, Ann-Kathrin Deisenhofer, Antonia Vehlen, Stephanie Vaccarezza Schürmann, Jessica Uhl, Danilo Moggia

**Affiliations:** 1 Department of Psychology, Trier University, Trier, 54296 Germany; 2 Doctoral Program in Psychotherapy, Pontificia Universidad Católica de Chile and Universidad de Chile, Santiago, Chile

**Keywords:** Outcome measurement, Precision mental health, Measurement-based care, Data informed psychological treatment, Treatment personalization

## Abstract

Outcome measurement including data-informed decision support for therapists in psychological therapy has developed impressively over the past two decades. New technological developments such as computerized data assessment, and feedback tools have facilitated advanced implementation in several seetings. Recent developments try to improve the clinical decision-making process by connecting clinical practice better with empirical data. For example, psychometric data can be used by clinicians to personalize the selection of therapeutic programs, strategies or modules and to monitor a patient’s response to therapy in real time. Furthermore, clinical support tools can be used to improve the treatment for patients at risk for a negative outcome. Therefore, measurement-based care can be seen as an important and integral part of clinical competence, practice, and training. This is comparable to many other areas in the healthcare system, where continuous monitoring of health indicators is common in day-to-day clinical practice (e.g., fever, blood pressure). In this paper, we present the basic concepts of a data-informed decision support system for tailoring individual psychological interventions to specific patient needs, and discuss the implications for implementing this form of precision mental health in clinical practice.

## Introduction

Decades of research on treatment effects have provided substantial evidence for psychotherapy as an efficient and cost-effective treatment for a wide range of psychological disorders (e.g., Barkham & Lambert, 2021; Cuijpers et al., 2023). Psychotherapy is also often superior to other interventions for psychological disorders such as psychopharmacological treatments (e.g., Hollon et al., 2021). This has led to a widespread acceptance and integration of psychotherapy within healthcare systems worldwide, and resulted in an increase in the establishment of standardized training regulations and certification requirements for therapists in numerous countries (e.g., Knox & Hill, 2021; Lutz, Castonguay et al., 2021).

However, psychological therapy is not a one-size-fits-all approach and responses can vary widely. This means that a patient beginning an evidence-based treatment with an average level of effectiveness may not necessarily achieve a successful outcome.

Therefore, in recent years, interest in patient-focused research and the concept of personalization or precision mental health has increased. The aim of this paper is to provide an overview of the state-of-the-art precision and personalization approaches that utilize data-informed strategies to facilitate clinical decision-making and tailor individual psychological interventions to specific patients.

## From Personalization to Precision Psychological Therapies

In this context, the idea of personalizing psychological therapies is not new, as therapists consistently engage in intuitive decisions aimed at providing the best treatment for each individual patient. However, the manner in which therapists approach this decision-making process to personalize treatments can vary significantly between practitioners (Cohen et al., 2021; Grove & Meehl, 1996; Lutz, de Jong et al., 2021). For instance, they can base their decisions on intuitive judgments, theoretical models, case conceptualizations, evidence from clinical guidelines, or a combination of these sources, among others. According to Cohen et al. (2021), it is possible to track a historical trajectory that reveals a developmental continuum of personalized treatment models, progressing from intuitive models, through theoretical to models informed by data and statistical algorithms.

Intuitive models can be considered an intuitive approach to clinical decision-making. These models entail therapists choosing treatment strategies and concepts based on their expert opinion, personal inclination, or familiarity. Thus, these intuitive models are influenced by the limitations of clinical judgment, which is often based on heuristics, biases, and clinical overoptimism (Ægisdóttir et al., 2006; Lilienfeld et al., 2014).

Traditional research and data-based concepts to personalization in psychological therapy have often been based on clinical guidelines that integrate findings from randomized controlled trials (RCTs) and meta-analyses on psychological treatments for specific diagnostic groups (e.g., defined by the Diagnostic and Statistical Manual of Mental Disorders − 5th Edition; APA, 2022). Here personalization entails aligning an evidence-based treatment with the patient’s primary presenting problem or diagnosis. In clinical practice, this leads to the prescription of therapies that have empirical support for treating specific clinical problems and populations.

When evaluating this classical research and data-based approach, it should be highlighted that the outcome derived from RCTs and meta-analyses is an average estimation of individual patient change trajectories observed among the participants of very heterogeneous groups. It is important to recognize that diagnostic groups are not homogeneous and defining patient subgroups by diagnostic category leads naturally to some heterogeneity in patient treatment trajectories. This variation has been investigated in several studies and is often referred to as the heterogeneity of treatment effects (Gabler et al., 2009; Kaiser et al., 2022). This heterogeneity is mostly neglected in RCTs and meta-analyses and individual characteristics that might be associated with differential treatment effects and therefore be relevant to tailor treatments remain overlooked.

As a result, in the last decade new models have been introduced that try to implement a more specific data-informed approach to personalize psychological therapy. This approach is based on predictive algorithms and feedback including a broader spectrum of patient characteristics (besides the diagnostic category) as well as larger data sets and more advanced statistical models (e.g., machine learning). By harnessing the power of extensive datasets, these approaches have the capability to offer clinical recommendations concerning the most suitable treatment for an individual patient (Lutz et al., 2020). In this regard, these approaches strive to provide precision psychological therapy in order to optimize treatment outcomes for each individual patient (e.g., Bickman, 2020; Brakemeier & Herpertz, 2019). To accomplish this objective, these approaches focus on identifying predictors of treatment response and the establishment of response profiles for subpopulations (Delgadillo et al., 2016). Different investigations into individual differences in patient characteristics encompasses various predictor variables, including sociodemographic variables, biomarkers, characteristics of symptom clusters, personality traits, behavioral phenotypes, and patterns of intrapersonal variability (e.g., Chekroud et al., 2021). The measurement of these characteristics in routine care serve as the foundation for algorithm-based recommendations, guiding clinicians in determining the treatment approach with the highest probability of yielding a positive therapeutic outcome for a specific patient (Chekroud et al., 2021).

## Treatment Selection Before Treatment Starts

As described above, before the onset of treatment, measurement-based and data-informed models might help to allocate patients to their optimal treatment option. This treatment selection process can be divided into four different components of treatment personalization: first, the selection of treatment approaches, packages, or protocols; second, the selection of treatment strategies, modules, or processes; third, the matching of patients to therapists; and fourth, employing intensive longitudinal assessments to support case conceptualization. All these approaches have a longstanding tradition, but have recently benefited from the rise of machine learning algorithms that can handle increasingly complex data from advanced assessments, modeling methods, and research designs (Delgadillo & Lutz, 2020, 2023).

## Treatment Approaches, Packages, and Protocols

Prognostic models can help to assign patients to their optimal treatment approach, package, or protocol, by applying several data-driven indices. These indices can be used as criteria for treatment selection. For example, patients having a poor prognosis in the Prognostic Index (PI) than usual, showed better outcomes if they received the high-intensity treatment intervention (cognitive-behavioral therapy, CBT) instead of the low-intensity treatment (Lorenzo-Luaces et al., 2017). If the prognosis was good, outcome did not differ between interventions. Besides the PI, several other indices have been applied to treatment selection in recent years, including the Leeds Risk Index (LRI; Delgadillo et al., 2016), the Expected Treatment Response (ETR; Lutz et al., 2006), and the Personalized Advantage Index (PAI; DeRubeis et al., 2014). Studies applying these indices have shown that receiving the recommended psychological therapy is associated with better patient outcomes. Another prognostic model recently developed by Cohen et al. (2023) relied on elastic-net regression for relapse prediction. The authors found that patients with the poorest prognosis benefited from switching to an alternative treatment during the course of therapy. Furthermore, the first prospective studies have already demonstrated the practical use of such prediction models (Delgadillo, & Atzil-Slonim, 2022).

**Fig. 1:**
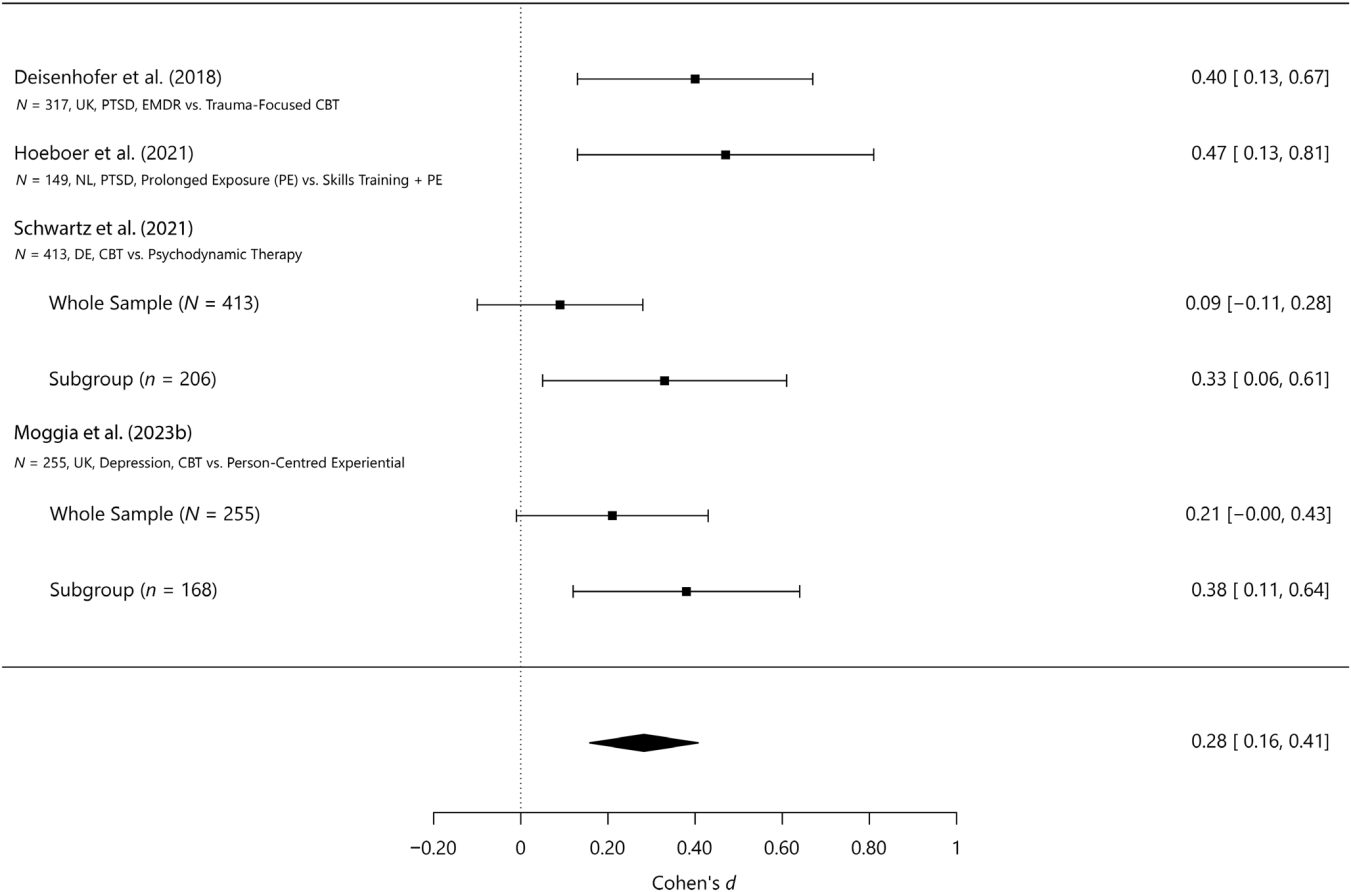
Forest plot of precision psychological therapy studies: outcomes of receiving the recommended treatment versus not receiving it. *Note*: CBT = cognitive-behavioral therapy. EMDR = eye movement desensitization and reprocessing. N = Sample size. NL = Netherlands. UK = United Kingdom. PTSD = Post-traumatic stress disorder

Figure 1 summarizes the results of several studies that developed prognostic models conducted with the involvement of our research group. A forest plot depicts the effect sizes of the difference in post-treatment scores between patients who received the treatment recommended by the models and those who did not. For instance, Deisenhofer et al. (2018) developed a prognostic model based on a sample of 317 patients from the UK diagnosed with Post-Traumatic Stress Disorder (PTSD) who received either eye movement desensitization and reprocessing (EMDR) therapy or trauma-focused cognitive behavioral therapy (Tf-CBT). The effect size of the difference in post-treatment scores between the recommended and the non-recommended treatment was *d* = 0.40 [0.13, 0.67]). Similarly, Hoeboer et al. (2021) estimated a model to recommend prolonged exposure (PE), intensified Prolonged Exposure (iPE), or skills training and PE for patients with PTSD. They applied it to a sample of 149 patients from the Netherlands, obtaining an effect size of *d* = 0.47 [0.13, 0.82]. In both of these studies, the models were tested retrospectively in the sample in which they had also been developed. Schwartz et al. (2021) developed a model to recommend either cognitive behavioral therapy (CBT) or psychodynamic therapy for patients with mixed diagnoses treated in the German public healthcare system (*N* = 1,379). The sample was split into training (70%) and holdout subsamples (30%) to develop and test the model, respectively. When the model was applied to the holdout sample (*n* = 413), a small and non-significant effect in the percentage change from pre- to post-treatment of *d* = 0.09 [–0.11, 0.28] was found. However, for the 50% of patients with the strongest recommendations, the effect size increased to *d* = 0.33 [0.06, 0.61]. Moggia et al. (2023b) estimated a model recommending CBT or person-centered experiential therapy for depression based on an RCT training sample of 255 patients from the UK. The model was evaluated in an independent routine care test sample (also comprising 255 patients) resulting in an effect size of *d* = 0.21 [–0.00, 0.43]. However, when the model was tested in a subgroup of patients (from the routine care test sample) with the strongest recommendations (at least a predicted effect size difference of *d* > 0.3), they showed a statistically significant difference in post-treatment scores of *d* = 0.38 [0.11, 0.64].

In summary, these studies all show incremental benefits for patients who received the treatments recommended by the prognostic models. A recent meta-analysis also confirmed that personalization is an effective strategy to improve outcomes in psychological therapy (Nye et al., 2023). However, the findings described above already demonstrate that the effect of treatment selection on outcome depends on the validation method. Once validation was conducted on a holdout sample of the same dataset (Schwartz et al., 2021) or even on a different external dataset (Moggia et al., 2023b), the effect was only observed in a subsample of patients with the strongest recommendations. In general, external samples are recommended for rigorous evaluation; however, when choosing them, it is essential to strike a good balance between similarity (to enable generalization) and dissimilarity (to assess generalizability to new data). Initial attempts suggest that the models might not always generalize as well as expected to new and potentially very different datasets (Moggia et al., 2023b; van Bronswijk et al., 2021). Furthermore, it is important to ensure that the test dataset has an adequate sample size for power issues (cf., Archer et al., 2021; Luedtke et al., 2019).

An even more rigorous test is the prospective application and validation of such models by assigning incoming patients to the treatment recommended by the algorithm and comparing this data-informed allocation to a random or clinically intuitive decision (Delgadillo et al., 2021; Lutz, Deisenhofer et al., 2022).

## Treatment Modules, Strategies, and Processes

Instead of selecting between treatment approaches or packages, it could be more beneficial to determine which evidence-based strategy (e.g., problem solving), module, or process within a treatment approach or package is the most promising for a given patient (e.g., Lutz et al., 2023; Ng et al., 2021; Wolitzky-Taylor et al., 2023; Zilcha-Mano et al., 2018). Therefore, process-outcome relations have also been used to select treatment components for individual patients.

For example, Rubel et al. (2020) introduced an approach to predict personalized process-outcome associations which have the potential to provide therapists with person-specific recommendations on which processes to focus on. Building on that study, Gomez Penedo et al. (2022) trained an algorithm to analyze cross-lagged effects of Problem-Coping Experiences (PCE) on outcome during the initial ten sessions of therapy. They found that the Random Forest algorithm was the most effective, explaining 14.7% of the process-outcome association in a training sample. Its results remained stable when predicting the same effect in the validation samples, explaining 15.4% of problem-coping effects on outcome. Similarly, Moggia et al. (2023a) conducted a study that focused on the association between resource activation, PCE and symptoms in CBT for depression on a sample of 715 patients. Continuous time dynamic modeling was used to model within-patient effects within the first ten sessions. The results showed significant cross-effects between the processes. While resource activation was recommended for patients with mild depression and high self-efficacy, PCE seemed more suitable for patients with severe depression and low self-efficacy.

In sum, these findings lead to a positive conclusion regarding the use of such models to personalize treatment strategies. However, it is a limitation of most studies in this area of research (such as those mentioned above) that the treatment recommendations were evaluated retrospectively. Lutz, Deisenhofer et al. (2022) conducted a prospective study of such process-outcome relations. They generated prediction models based on a large archive sample of 1,234 already-treated patients and applied them to a new sample to recommend patient-specific optimal treatment strategies (problem-solving, motivation-oriented, or mixed strategy) for the first ten sessions (for further information see the section on The Trier Treatment Navigator).

## Precision and Patient-Therapist Matching

Numerous studies in psychological therapy have demonstrated the variance in treatment outcomes across therapists, also known as therapist effects (Baldwin & Imel, 2013). Despite using the same treatment protocol, therapist variation is an important factor to consider in treatment planning, while some therapists achieve impressive clinical outcomes, others seem less effective. Therapist effects are partially associated with patient features, as particularly effective therapists prove especially beneficial for patients with severe levels of distress and risk of self-harm compared to their less effective colleagues (Saxon & Barkham, 2012). Therapists with the ability to work effectively with highly challenging cases are more likely to produce positive outcomes, and interpersonal skills seem to be a pivotal factor to explain differences between more and less effective therapists (Anderson et al., 2016; Heinonen & Nissen-Lie, 2020). Matching patients to specific therapists based on their demographic and clinical features has been shown to be a potentially effective method of treatment allocation. Recent research indicates that certain patients respond better to therapy with specific therapists, and archival clinical data can be used to predict the likely outcome of new patients assigned to specific therapists, potentially facilitating an evidence-based approach to patient-therapist matching (Constantino et al., 2021; Delgadillo et al., 2020).

## Precision and Intensive Longitudinal Assessments

In order to improve predictive accuracy and treatment recommendations, recent studies have begun employing intensive longitudinal or ecological momentary assessments (EMA; e.g., Fisher et al., 2019; Lutz et al., 2018; Webb et al., 2022). This approach utilizes smartphones or other electronic devices to repeatedly inquire about patients’ symptoms, activities, thoughts, or emotional experiences throughout the day (e.g., four times per day) for a defined period of time (Shiffman et al., 2008). By using EMA data, personalized treatment plans can be drawn up for patients, taking into account their specific symptom profiles. Through the use of techniques such as network analysis, associations between patient symptoms can be identified and visualized (e.g., Bringmann et al., 2022). In the network concept, it is assumed that symptoms interact with and sustain one another, rather than being attributable to a latent underlying disease factor (Borsboom, 2017; Hofmann & Hayes, 2019; Wright & Woods, 2020). This information can then be used to identify core symptoms in the patient’s network, highlighting potential targets for treatment with an increased chance of symptom reduction (Hofmann & Hayes, 2019). Procedures to translate individual network models into personalized treatment that focuses on core symptomatology have already been introduced (Rubel et al., 2018), and an initial prospective study of 40 patients was able to demonstrate the value of using EMA data to predict treatment (Fisher et al., 2019). However, the study’s scope was constrained by the absence of a treatment-as-usual (TAU) control group, which restricts the extent to which conclusions can be drawn concerning the incremental advantages of this approach. Furthermore, network and alternative time-series models have found application in forecasting treatment dropout (Lutz et al., 2018), as well as recovery from major depressive disorder or patient-specific interventions (Fisher et al., 2019). First studies have also been conducted to test the implementation and use of EMA data to monitor treatment progress (e.g. Bos et al., 2022).

## Treatment Decisions after Treatment Starts: Measurement-Based Care, Practice-Oriented Research, Patient-Focused Research and Practice-Based Evidence

The integration of empirical data to support clinical decision-making described in the previous sections has the potential to advance the field of psychological therapy. This is especially the case, if it is not only applied at the very beginning of treatment, but extended by routine assessments and feedback to therapists throughout therapy. To effectively develop and implement such systems in clinical practice, it is crucial to establish a measurement-based framework and incorporate continuous feedback into clinical care. This approach has been dubbed practice-oriented research, patient-focused research, practice-based evidence, and routine outcome monitoring (ROM; Barkham & Lambert, 2021; Castonguay et al., 2013; Castonguay, 2021; Lutz et al., 2021a). It is based on data assessed before and after, but also during treatment with varying frequencies: (a) Pre–post assessments, which are easy to implement, but only allow the consideration of simple changes with a high probability of missing data; (b) repeated measurements throughout treatment, which can inform treatment decisions based on psychometric feedback on linear and non-linear patterns of change; (c) session-by-session assessments, whose even higher resolution allows individual treatment progress to be tracked continuously, triggering clinical support tools provided to therapists for treatment adaptation as needed. A large body of research including several meta-analyses for different problem areas and services show the positive effects of ROM on treatment progress and outcome, especially for patients at risk for treatment failure (e.g., Barkham et al., 2023; Bickman, 2020; de Jong et al., 2021; Lutz, Deisenhofer et al., 2022). In the following, we will dive deeper into the measurement-based and data-informed models that extend beyond the beginning of treatment to ongoing assessment, monitoring, and adaptation throughout the therapeutic process.

## Treatment Monitoring and Adaptation

Tracking and assessing patient progress, i.e., monitoring treatment, helps therapists to evaluate treatment effectiveness, to make empirically-informed adjustments as needed, and to ensure that patients are receiving the most effective care possible. ROM comprises short self-report questionnaires to measure outcomes continuously throughout treatment. Feeding this psychometric information back to therapists enables them to evaluate whether their current approach has been successful or whether adaptations are necessary (e.g., Barkham et al., 2023; Lutz, Rubel et al., 2022). This process is implemented via computerized feedback systems that offer decision rules based on empirical data (i.e., datasets from clinical practice settings). These rules help therapists identify whether a patient is improving or at risk of treatment failure and can guide them to make treatment decisions that are tailored to the patient’s specific needs and goals.

A substantial body of evidence (over 40 randomized clinical trials and numerous meta-analyses) supports the use of ROM and feedback in clinical practice. This research has shown that treatments incorporating feedback lead to better outcomes, lower dropout rates, and greater efficiency compared to standard evidence-based treatments. The latest and most comprehensive meta-analyses found that ROM and feedback had a significant advantage over treatment as usual, with effect sizes of *d* = 0.14 – 0.15. These effects were larger for patients who did not initially respond to treatment, i.e., those who were at risk of treatment failure, with effect sizes of *d* = 0.17 – 0.29 (de Jong et al., 2021; Rognstad et al., 2023). In addition, feedback has been shown to have a small favorable effect on dropout rates (*OR* = 1.19).

It is important to consider two factors when assessing the effects of ROM and feedback. Firstly, these effects are in addition to those produced already by evidence-based treatments the patients are already receiving. Secondly, feedback is a low-cost technological intervention that does not impose a large burden on patients and therapists. A large randomized controlled trial involving 2,233 participants showed that adding ROM and feedback to evidence-based psychological treatments in the UK Improving Access to Psychological Therapies (IAPT) system was cost-effective. While feedback was associated with a non-significant increase in costs per case (£15.17), it helped 8.01% more patients to be reliably improved at the end of the treatment (Delgadillo et al., 2021). In addition, research has shown that the effectiveness of feedback can be further enhanced by using additional clinical support tools (Lambert et al., 2018).

Nevertheless, not all therapists experience improved outcomes when using feedback, which seems to be due to differences in therapists’ attitudes towards these systems and how they utilize the feedback information. Therefore, feedback and evidence-based decision rules are now considered crucial clinical competencies and a significant part of training to ensure correct implementation and application of these systems (e.g., Lutz, de Jong et al., 2021).

## The Trier Treatment Navigator

The Trier Treatment Navigator (TTN; Lutz et al., 2019) is an example of a comprehensive decision and navigation system that utilizes modern technology to integrate data-informed, measurement-based care, while giving clinicians the freedom to apply the algorithm-based recommendations or to overrule them if necessary. The TTN supports therapists with an algorithm-based selection of treatment strategies and enables them to make informed adaptations over the course of therapy. During their clinical training at the University of Trier’ outpatient center, therapists are trained and encouraged to use the feedback system, which is regarded as an evidence-based decision-support system that complements the clinical perspective. The TTN integrates socio-demographic data as well as process and outcome measures to provide a more comprehensive understanding of the patient’s needs. It comprises session-by-session process and outcome measures and thus monitors treatment progress. In the following, we will describe how the TTN can support personalized treatment selection, outcome monitoring, and personalized treatment adaptations.

**Fig. 2:**
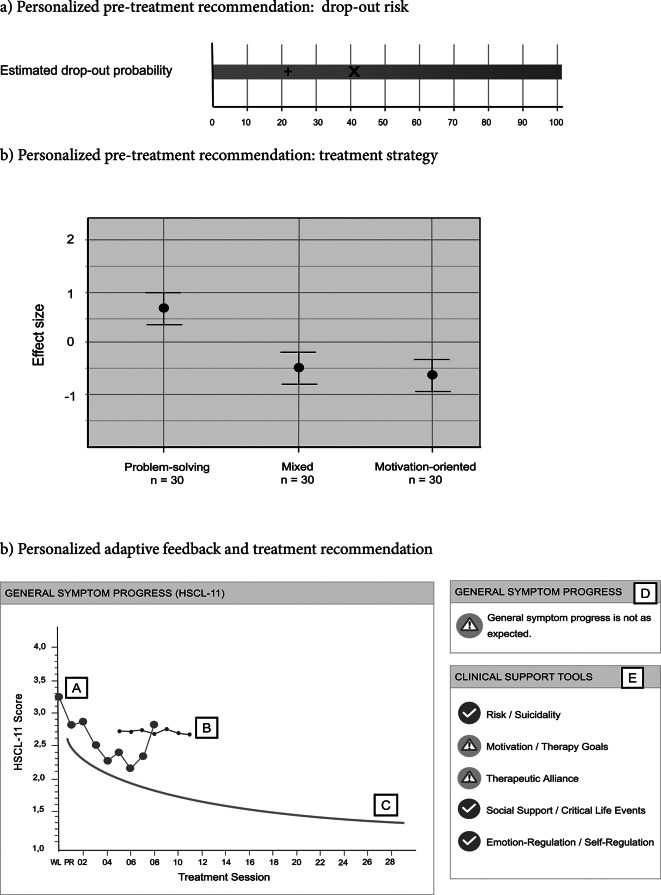
The interface of the Trier Treatment Navigator (TTN), showing the pre-treatment recommendations, monitoring, and treatment adaptations. *Note*: In b), line (A) indicates the actual symptom severity, line (B) represents the individual risk value, line (C) represents the expected course of symptom severity. On the right-hand side, the warning sign (D) and the domains relevant to the current problem are displayed (E). For this case, especially the domains “Motivation/Therapy Goals” and “Therapeutic Alliance” seem to be affected. HSCL, Hopkins Symptom Checklist; PR, pretreatment; WL, waitlist

At the onset of treatment, therapists receive an overview of their patients’ potential problem areas, including risk of suicidality, substance abuse, treatment expectations, symptomatology, and interpersonal issues. The TTN calculates each patient’s individual dropout risk based on multiple predictors derived from prior studies on dropout probabilities, such as initial impairment, interpersonal functioning, personality traits, and treatment expectations. The individual dropout risk is then compared to the outpatient clinic’s average dropout risk (see Fig. 2a) so that therapists can include this information in their case formulations (e.g., Moggia et al., 2022; Schaffrath et al., 2022).

The TTN provides a personalized treatment recommendation for the first ten sessions, using a vast archival dataset that includes therapist reports on whether a more motivation-oriented, problem-oriented, or mixed strategy was employed. The nearest-neighbor method is utilized to identify the most comparable patients who received treatment with these approaches, based on specific variables that significantly correlate with pre-to-post improvement, and an effect size is computed for each approach based on these similar subsamples. Therapists are provided with graphical results, allowing them to visualize which approach is more likely to yield the best treatment outcome for their patient (see Fig. 2b). Both pre-treatment recommendations support therapists in their intuitive decision-making process by providing additional information.

During treatment, patient symptoms are assessed in each session and reported back to therapists (see Fig. 2c). Furthermore, in addition to the patient’s individual values, the TTN calculates an expected change curve based on the most similar patients who have previously undergone treatment at the outpatient clinic. After the first four sessions, a risk value is determined for each patient, in comparison to similar patients, above which the likelihood of a negative outcome is increased. This enables the therapist to compare the patient’s actual symptom severity course with the predicted course of improvement. If symptom severity exceeds the risk value, a warning symbol is displayed in the digital feedback portal and reported back to the therapist. Additionally, the Affective Style Questionnaire (ASQ; Hofmann & Kashdan, 2010) and the Assessment for Signal Clients (ASC; Lambert et al., 2007) are utilized every five sessions to cover five domains related to the therapy process: crisis management/suicidality, motivation/therapy goals, therapeutic alliance/interpersonal skills, social support/life events, and emotional/self-regulation ability. A green signal is displayed if everything is progressing as expected and the patient is improving. However, if the patient’s responses to the questionnaires exceed a cut-off value, an orange warning signal is also displayed, indicating high impairment in these domains. Therapists can click on the warning signal to obtain more information on the specific domain and clinical problem-solving tools that offer extensive evidence-based material, such as worksheets, video examples, and instructions for exploring and addressing the problematic situation. These support tools are an extension of the clinical support tools developed by Lambert (e.g., 2007). Therapists can also access deliberate practice videos on various problem areas, in which actors simulate patients in challenging therapy situations. Therapists can train their skills by responding to the video. In this way, the TTN supports the personalized and evidence-based adaptation of ongoing therapy.

A randomized controlled trial involving 538 patients was carried out to prospectively evaluate the TTN system (Lutz, Deisenhofer et al., 2022). Therapist-patient dyads were randomized after their initial screening to either have access to the TTN system or not. The study was based on a crossed-therapist design, meaning therapists treated patients in both conditions. The study revealed that following the recommended treatment strategy in the first ten sessions resulted in an effect size increase of about 0.3. Additionally, linear mixed model analyses demonstrated that therapist symptom awareness, attitude, and confidence in feedback were significant predictors of outcome. The usefulness of feedback rated by therapists was also found to be a significant moderator of the feedback–outcome and not-on-track–outcome associations. All in all, the TTN can support therapists in their intuitive decision-making by providing data-driven recommendations for data-informed treatment strategies. However, these results highlight the importance of prospective studies and high-quality implementation of the TTN system in clinical practice, as the effects are highly dependent on therapists’ perceptions of its usefulness.

## Summary

Tailoring treatments to patients has always been a key area of psychotherapy research. This paper summarizes the historical development of precision mental health – covering intuitive and ‘informal’ concepts as well as current data-driven and data-informed approaches including the tailoring of treatment interventions at the beginning as well as during treatment.

Data-informed approaches are empirically-based and can therefore be considered precision approaches to personalization tasks in mental health, similar to the precision health concepts in medicine (e.g., Bickman, 2020; Lutz et al., 2022). Precision mental health utilizes predictive algorithms rooted in statistical models, machine learning, and artificial intelligence to systematically consider the individual differences between patients. It also emphasizes the importance of ongoing treatment monitoring and adaptation. Leveraging extensive datasets, these approaches aim to offer clinical recommendations for the most suitable treatment for individual patients, especially for patients at risk for treatment failure. Thereby, they optimize treatment outcomes and resource allocation, reducing dropout. Of course, this assumes well-tested algorithms (including prospective evaluations) and secure data implementations.

Despite the benefits, not all therapists experience improved outcomes with such algorithm-based systems, as ROM research shows. Additional improved multi-cultural adaptations of algorithms are necessary and data-informed approaches must be tested in several representative samples across diverse clinical settings. Their success depends on if and how therapists utilize the feedback information. Therefore, the skill of using such feedback from data-informed systems and evidence-based decision rules must be considered a crucial clinical competency and an essential part of clinical training.

Data-informed or precision approaches to personalization, while valuable in many ways, are not without their disadvantages. One significant drawback should be mentioned: Special training and care are needed to ensure that therapists do not blindly follow the system’s suggestions, but rather use the system responsively by reevaluating it based on their clinical knowledge. It is crucial that therapists remain responsive, adaptable, and focused on the individual patient’s needs and experiences, while simultaneously making use of the best empirical information available. Therefore, it is crucial that therapists remain responsive, adaptable, and focused on the needs and experiences of the individual patients (e.g., Stiles, 2017). A balanced integration of data-informed insights with therapist expertise, skills and patient experiences can improve the quality of practice. Integrating data-driven algorithms into clinical practice does not invalidate the significance of traditional approaches to personalization. Instead, it enhances the overall landscape of clinical practice by offering valuable insights and augmenting decision-making processes.

The Task Force on Evidence-Based Practice of the American Psychological Association (APA, 2006) defines evidence-based practice in psychology as “(…) the integration of the best available research with clinical expertise in the context of patient characteristics, culture, and preferences” (p. 273). That is to say, clinical decision-making should be based on the best available research evidence, and at the same time, clinicians should use their own clinical expertise and consider the patient’s features, culture, and preferences to make a decision on treatment selection and adaptation. However, usually, clinical guidelines base their clinical recommendations exclusively on the available evidence coming from RCTs and meta-analyses, and the other two dimensions (clinicians’ expertise, and patients’ individual characteristics, preferences and culture) remain neglected (e.g., Aubel & Chibanda, 2022). It may be apparent to the reader that our discussion has led us to a conceptualization of a broader model and that the best available research evidence does not necessarily come from RCTs, meta-analyses, and clinical guidelines, but additionally from data-driven algorithms including a broader set of patient characteristics as the basis for decision-making.

One example of how data-informed personalization can be integrated into clinical practice is the Trier Treatment Navigator, featuring pre-treatment recommendations, treatment monitoring, and adaptation. An essential aspect of this feedback system is that it must be used by scientifically trained therapists who can integrate the data-driven recommendations into their evidence-based disorder and treatment knowledge, their theoretical approach, and clinical experience to make the best possible treatment decision.

To conclude, data-informed approaches can support and extend evidence-based practice and should be integrated as part of the clinical decision-making process and implemented into clinical training. The integration of this approach can increase the effectiveness and efficiency of patients’ treatment, especially for those at risk for a negative treatment outcome. Feedback systems like the TTN are the products of decades of research and it is not necessary for every individual therapist to immediately install such a comprehensive navigation tool. An initial step toward data-informed personalization in clinician practice could be the introduction of continuous measurement and the attempt to use this psychometric information in treatment. Future research should focus on several aspects. One focus could be identifying strategies to increase acceptance and implementation of such feedback systems into clinical practice (e.g., Douglas et al., 2023). Another is to continue improving data-driven recommendations to tailor treatments at the beginning as well as throughout treatment (e.g., Lutz et al., 2022). For example, multimodal findings on psychological distress and dynamic changes in the initial phase of therapy can be integrated into empirically supported case conceptualization and therapy planning. The systems themselves could also be developed further by investigating new machine learning algorithms and prediction models as well as improving the decision-making process during the course of treatment. Furthermore and most importantly, more prospective studies should be conducted to test the new models and compare them with traditional case conceptualization in a variety of settings and cultures, including minority groups and underserved populations.
